# Effects of elevated temperature on gene expression, energy metabolism, and physiology in brown trout, *Salmo trutta*

**DOI:** 10.1093/conphys/coaf025

**Published:** 2025-04-23

**Authors:** Buumba Hampuwo, Anna Duenser, Franz Lahnsteiner

**Affiliations:** Federal Agency for Water Management, Institute for Water Ecology, Fisheries and Lake Research, Scharfling 18, A-5310 Mondsee, Austria; Institute of Hydrobiology and Aquatic Ecosystem Management (IHG) 1180 Wien, Gregor-Mendel-Straße; Federal Agency for Water Management, Institute for Water Ecology, Fisheries and Lake Research, Scharfling 18, A-5310 Mondsee, Austria; Federal Agency for Water Management, Institute for Water Ecology, Fisheries and Lake Research, Scharfling 18, A-5310 Mondsee, Austria

**Keywords:** brown trout, elevated temperature, gene expression, metabolites, energy reserves

## Abstract

Given the imminent threat of global warming and rising water temperatures in Austria, this study investigated the effects of elevated temperature on gene expression, energy reserves, and cellular energy status in brown trout (*Salmo trutta*), a species particularly sensitive to increasing water temperature. A total of 250 fish were placed in four stream channels under flow-through conditions. Two channels were maintained at 9 °C as controls, while the other two had their temperature gradually increased to 20 °C over seven days and then maintained at 20 °C for 21 days. Sampling was conducted on day 1, after the temperature reached 20 °C, and the last day of high-temperature exposure on day 21. At each sampling point growth, hepatosomatic index and the fat content of the viscera were measured and/or calculated, and liver samples were taken for gene expression and metabolite analyses. Elevated temperature significantly increased the expression of genes related to cellular stress response (*hsp70*, *hsp90 aa1*, *cat*, and *casp8*) compared to controls. However, there was no significant difference in the expression of genes associated with lipid and carbohydrate metabolism (*d5fad* and *pfkfb4*). Furthermore, there was a decrease in energy storage indicated by a decrease in the hepatosomatic index, glycogen, triglycerides and ATP in the liver as well as the fat content of the viscera. Cellular energy status also significantly decreased, as indicated by the calculated adenylate energy charge. Physiologically, this culminated in suppression of growth in the treatment group after 21 days. This study shows that elevated temperature leads to significant trade-offs in brown trout, which may lead to ecological consequences over the long run. These findings offer critical insights into the physiological impacts of elevated temperature that help evaluate the species' acclimation to rising water temperature and inform the development of effective conservation strategies in a warming world.

## Introduction

Global warming is a major threat to aquatic ecosystems, particularly to freshwater ecosystems in temperate regions. The Intergovernmental Panel on Climate Change (IPCC) special report (2018) has stated that a 1.5 °C to 2 °C increase in global temperature will have a significant impact on aquatic ecosystems. These consequences include species relocating to higher elevations and the extinction of those that are unable to adapt. According to the National Oceanic and Atmospheric Administration (2019), water temperatures are projected to rise even faster as we approach 2100. Consequently, Filipe *et al*. (2013) estimate that by 2080, over 64% of European rivers will become uninhabitable for brown trout, one of the most vulnerable species within the Salmonidae family.

Compared to other riverine salmonids, such as rainbow trout (*Oncorhynchus mykiss*), brook trout (*Salvelinus fontinalis*), and Atlantic salmon (*Salmo salar*), brown trout (*Salmo trutta*) has noticeably narrower temperature limits. Rainbow trout, brook trout, and Atlantic salmon can tolerate a lower critical temperature of 0 °C and a chronic upper temperature of 30 °C (Elliott & Elliott, 2010; Wehrly *et al*., 2007; Jonsson & Jonsson, 2009). In contrast, brown trout's lower critical temperature is 4 °C, and stress begins at an upper temperature of 20 °C (Elliott, 1981; Jonsson & Jonsson, 2009; Nudds *et al*., 2020). However, it is important to note that the mode of exposure to critical temperatures, the magnitude of the temperature, and the exposure time determine the species' response. Acute exposure typically results in low tolerance and mortalities, whilst a gradual decrease or increase in temperature usually results in acclimatisation (Adams *et al*., 2022; McKenzie *et al*., 2021).

Water temperature plays a critical role in regulating the physiological processes of fish, including gene expression, energy metabolism, and energy storage (Lou *et al*., 2019). As obligatory poikilothermic organisms, fish undergo changes in body temperature in direct response to fluctuations in their aquatic environment, which in turn affects their survival, growth, and disease resistance (Pörtner & Farrell, 2008; Jobling, 1993; Harvell *et al*., 2002; Johnston & Dunn, 1987). The influence of water temperature on energy metabolism and storage arises primarily from elevated temperatures increasing metabolic rates and energy demands, leading to shifts in energy allocation and utilisation (Kullgren *et al*., 2013; Brown *et al*., 2004). Similarly, water temperature has a pronounced effect on gene expression, with studies reporting widespread transcriptional changes across various tissues. These responses often involve genes linked to cellular stress response (CSR), including those that regulate heat shock response, oxidative stress, apoptosis, and metabolic processes (Jeffries *et al*., 2012; McBryan *et al*., 2013; Olsvik *et al*., 2013; Rebl *et al*., 2013; Li *et al*., 2024; Beemelmanns *et al*., 2021; Huang *et al*., 2018; Wade *et al*., 2019; Bo *et al*., 2023; Chen *et al*., 2023; Han *et al*., 2023; Ma *et al*., 2023; Shi KunPeng *et al*., 2019).

Given this background of the known effects of temperature on fish, this study aimed to explore whether similar responses are elicited in brown trout in order to gauge acclimatisation processes and understand the ecological implications on the species in the face of rising water temperature in Austria. Therefore, brown trout fingerlings were exposed to an elevated temperature of 20 °C for 21 days, a threshold identified as the upper limit inducing stress in this species (Elliot, 1981). This temperature is also ecologically significant, as long-term monitoring in Austria has documented rising water temperatures from 1984 to 2004, with measurements from 76 stations showing some summers exceeding 20 °C (Prinz *et al*., 2009; Melcher *et al.,* 2013).

Specifically, the present study assessed the effects of elevated temperature on the expression of genes associated with the heat shock response (*hsp70* and *hsp90aa1*), oxidative stress (*cat*), apoptosis (*casp8*), lipid and carbohydrate metabolism (*d5fad* and *pfkfb4*, respectively). In addition, we evaluated energy metabolism by measuring key metabolites glycogen and triglycerides which are critical energy reserves in the liver. ATP, ADP, and AMP were also measured in the liver, which provides insights into cellular energy status. Finally, we investigated energy storage by analysing the changes in the hepatosomatic index and the fat content of the viscera across treatments, and to better understand the overall physiological effects of elevated water temperature on brown trout, we compared the growth rate across treatment groups.

We hypothesised that chronic exposure to elevated water temperatures in brown trout causes an increase in the expression of cellular stress response genes (*hsp70*, *hsp90aa1*, *cat*, and *casp8*) and a reduction in metabolic-related genes, as the reregulation of their expression may help mitigate the effects of heat stress. We also hypothesised that elevated temperature leads to a reduction in energy metabolites and overall cell energy status as energy is redirected toward cellular stress responses.

As far as we know, the combined effect of elevated temperature on gene expression and energy metabolism in brown trout has not been previously investigated. Despite several studies having been conducted to assess these factors independently in closely related species, such as rainbow trout and Atlantic salmon (Lahnsteiner, 2022; Auer *et al*., 2015; Millidine *et al*., 2009; Closs & Lake, 1996), research on brown trout is missing.

## Materials and Methods

### Ethical declarations

Experiments were carried out in accordance with Austrian regulations governing animal welfare and protection and with the EU directive 2010/63/EU for animal experiments. Furthermore, the study was also approved by a committee of the Federal Agency for Water Management and of the Federal Ministry for Agriculture, Forestry, Regions and Water Management. It is officially listed as Project 3143.

### Experimental set-up

250 brown trout fingerlings (total length 7.9 ± 0.5 cm, body mass 4.9 ± 0.7 g) previously acclimated to 9 °C during their whole life cycle and exposed to a natural photoperiod were stocked in four stream channels (190 x 25 x 35 cm, length x width x height) under flow-through conditions (0.2 L·s^-1^). Two stream channels were maintained at 9 °C and served as control. In the other two tanks, the temperature was gradually raised to 20 °C for seven days using a geothermal heat pump and kept at 20 °C for 21 days.

### Water quality parameters

Groundwater was used in the experiment, and water quality parameters were measured daily using a digital multimeter 3630 IDS SET G (Xylem Analytics, Weilheim, Germany). The recorded values (mean ± SD) included an oxygen saturation of 98.4 ± 1.6%, pH ranged from 7.86 to 8.01 ± 0.01, and conductivity of 341.5 ± 8.0 μs cm^-1^ at 25 °C. The acid-neutralising capacity (ANC) was determined using the titration method (Weber *et al*., 2005) and measured at 3.35 ± 0.02 mval l^-1^. Phosphate (PO₄^3-^) and ammonium (NH₄^+^) concentrations were below 0.005 mg l^-1^, as analysed following the method described by Fresenius *et al*. (2011). These parameters remained consistent across both temperature conditions (9 °C and 20 °C).

Dissolved oxygen (DO) concentration was 11.59 ± 0.21 mg l^-1^ in the 9 °C control group and 8.16 ± 0.25 mg l^-1^ in the 20 °C treatment group. These water quality parameters provided optimal conditions for the fish, ensuring that any variation in results would not be attributed to suboptimal water quality conditions but to temperature differences alone (Baur & Rapp, 2003).

### Sampling, growth, and condition indices

Sampling was conducted twice for the control and treatment group, specifically on day one after a gradual water temperature increase to 20 °C (treatment beginning, TB) and the last day of high-temperature exposure on day 21 (treatment end, TE). Sampling for the control group was conducted at the same time points as for the treatment group (control beginning, CB; control end, CE).

On each sampling occasion, ten fish were randomly selected from each of the four tanks. The body mass of the fish was measured, and the liver of each fish was extracted and weighed to calculate the hepatosomatic index [HSI = Mass of liver/body mass * 100]. Thereafter, a portion of the liver was weighed and stored in perchloric acid for metabolite analysis and a separate portion was stored in RNAlater for gene expression analysis. The viscera were also excised and kept at -20 °C for later determination of fat content following the chloroform-methanol method described by Bligh and Dyer (1959). Finally, the per cent survival at the end of the experiment for each treatment (S%) was calculated using the equation: S% = [Number of Fingerlings at end of experiment/Number of Fingerlings stocked*100].

### RNA extraction, cDNA synthesis and qPCR

Five fish from each sampling occasion were used. For RNA extraction, 3 mg to 5 mg of liver samples, previously stored in RNAlater at -20 °C, were utilised, following the protocol provided by the manufacturer of the commercial kit (NucleoSpin® RNA Plus kit, Macherey-Nagel, Düren, Germany). Five biological replicates were analysed for each sampling point. The quantity and quality (IQ) of the extracted RNA were determined using the Invitrogen Qubit^TM^ 4 Fluorometer with the respective Qubit^TM^ assay kit (Thermo Fisher Scientific, Vienna, Austria), and only RNA with an IQ Score exceeding 7 was used for cDNA synthesis.

For cDNA synthesis, the sample volumes were adjusted to yield a constant concentration of 1000 ng RNA. cDNA synthesis was performed using the Thermo Scientific RevertAid First Strand cDNA Synthesis Kit (Thermo Scientific, Schwerte, Germany), following the manufacturer's protocol.

Primers for Real-time PCR in this present study were adopted from earlier studies of brown trout or closely related species as cited in Table 1 and were synthesised by Sigma-Aldrich (Merck KGaA, Darmstadt, Germany). Primer efficiency was determined for each gene using standard curves that were generated by serial dilutions of pooled cDNA. Standard curves were run in duplicates and calculated with the formula E= -1+10^(-1/slope), and only primers with an amplification efficiency above 90% were used.

**Table 1:** Primers used for qPCR analysis.

Gene	Primer Forward 5´→3´(Tm°)	Primer Reverse 5´→3´ (Tm°)	Amplicon Length BP	Primer Efficiency	Reference
*hsp70*	AGTGATCAACGACTCGACACG (65.4°C)	CACTGCATTGGTTATAGTCTTG (58.8°C)	121	93%	Hansen *et al*., 2006
*hsp90aa1*	CGAGGACATGAAGAAGAGGCAT (66.6°C)	ACACTGTCACCTTCTCCACTTT (61.6°C)	98	105%	Meier *et al*., 2014
*cat*	CGACGATAACGTCACACAGG (62.3°C)	GCCTGGACCCCGTTTCCATA (70.0°C)	177	101%	Beemelmanns *et al*., 2021
*casp8*	TCCTGTCTATATAAGTGGGCGTTC (64.0°C)	CTTTCCCGAGTGAGCTAACAGT (63.1°C)	141	91%	Beemelmanns *et al*., 2021
*delta-5fad*	GTGAATGGGGATCCATAGCA (64.6°C)	AAACGAACGGACAACCAGA (63.29°C)	192	96%	Morais *et al*., 2011
*pfkfb4*	CACGGAAGTGAAAGTGAGCA (64.1°C)	CGTCCAAAGGCACATAGGTT (63.8°C)	113	100%	Caballero-Solares *et al*., 2018
*gapdh*	AGGCATCTCACAAACGAGGA (64.8°C)	GGCAACAATCTCAACTCCCT (63.0°C)	80	92%	Meier *et al*., 2014

Melting temperature Tm°, base pairs BP.

Real-time PCR was performed in duplicates on a qTOWER3 thermal cycler (Analytik Jena, Jena, Germany). Ct values were determined with the software qPCRsoft 4.0. Each gene was quantified relative to the expression of a housekeeping gene, *gapdh,* using the Pfaffl (2001) method. Expression levels are presented as log_2_-fold change relative to the average expression levels of the control group.

### Measurement of metabolites

All chemicals and enzymes used for the assays were analytical grade and obtained from Sigma-Aldrich (Merck KGaA, Darmstadt, Germany). Ten fish from each sampling occasion were used. To obtain a reference unit for metabolic measures, liver subsamples were weighed in 1.5ml microcentrifuge tubes with an analytical balance (Mettler Toledo, Vienna, Austria). The metabolites were extracted with 3 mol l^−1^ perchloric acid. Samples were homogenised and kept in the extraction fluid for 20 minutes under constant agitation, then the homogenate was centrifuged at 1500 g for 10 min, and finally, the supernatant was collected and neutralised with 1 mol l^−1^ Na_2_CO_3_. Energy storage metabolites glycogen and triglycerides and cell energy status metabolites ATP, ADP and AMP were measured using routine UV spectrophotometric assay, as described in methods for enzymatic analysis by Bergmeyer (1985). Measurements were made with a Multiskan FC microplate reader at 340 nm (Thermo Fisher Scientific, Vienna, Austria). Briefly, the metabolite assays included the following chemicals and enzymes:

Glycogen levels were determined by measuring the glucose released after glycogen digestion with amyloglucosidase (54,000 U/L) in a sodium acetate buffer (118 mmol l^-1^, pH 4.5). The glucose assay used a working solution consisting of triethanolamine buffer (375 mmol l^-1^, pH 7.6), MgSO₄, (8 mmol l^-1^), NADP^+^ (0.75 mmol l^-1^), glucose-6-phosphate dehydrogenase (16,000 U/L), and hexokinase (7,400 U/L).

Triglyceride levels were measured indirectly by the glycerol released during triglyceride hydrolysis. A working solution was prepared using potassium buffer (100 mmol l^-1^, pH ~7.1) and lipase (1,000 U/L) to hydrolyse triglycerides. The glycerol assay was performed using the same buffer system containing MgCl_2_ (3 mmol l^-1^), ATP (0.35 mmol l^-1^), phosphoenolpyruvate (0.5 mmol l^-1^), NADH (0.2 mmol l^-1^), glycerol kinase (2,000 U/L), lactate dehydrogenase (LDH, 2,000 U/L), and pyruvate kinase (2,500 U/L).

AMP and ADP were analysed simultaneously by NADH oxidation. A working solution consisting of triethanolamine buffer (750 mmol l^-1^, pH ~7.5), MgCl₂ (58 mmol l^-1^), KCl (101 mmol l^-1^), NADH (0.48 mmol l^-1^), phosphoenolpyruvate (2.25 mmol l^-1^), lactate dehydrogenase (125000 U/L), and pyruvate kinase (PK, 2,500 U/L) was used for ADP analysis. After the reaction was terminated myokinase (3,000 U/L) was added to the working solution for AMP determination.

For ATP determination, a reaction mixture containing triethanolamine buffer (750 mmol l^-1^, pH ~7.5), MgCl₂ (210 mmol l^-1^), NADP (10 mmol l^-1^), glucose (550 mmol l^-1^), glucose-6-phosphate dehydrogenase (16000 U/L) and hexokinase (7400 U/L) was used.

Finally, the adenylate energy charge (AEC) was calculated, providing further information on cellular energy status using Formula 1 from Ivanovici (1980).


(Formula 1) \begin{equation*} \mathbf{AEC}=\frac{\mathbf{Conc}\ \mathbf{ATP}+\mathbf{0.5}\ \mathbf{x}\ \mathbf{Conc}\ \mathbf{ADP}}{\mathbf{Conc}\ \mathbf{ATP}+\mathbf{Conc}\ \mathbf{ADP}+\mathbf{Conc}\ \mathbf{AMP}} \end{equation*}

### Statistical analysis

Statistical analyses were conducted in SPSS (version 23). Normality was checked using the Shapiro-Wilk test, and homogeneity of variance was assessed with Levene's test. For data that met the normality assumptions (Body mass, HSI and FCV), one-way analysis of variance (ANOVA) was used to compare means across groups. Then, the post hoc Tukey test was used to determine which group was statistically different.

On the other hand, the metabolite data did not meet the assumptions required for parametric analysis; therefore, the *Kruskal Wallis H* test was used to test for statistical differences between the three groups. To determine which group was statistically different, a post hoc *Dunn* test was conducted.

Gene expression data was first log_2_-transformed to aid in calculating fold change and have a near-normal distribution. Despite log transformation, the parametric assumption was not met. Therefore, the *Kruskal Wallis H* test was used to test for significant differences, followed by the post hoc *Dunn* test. All statistical analyses were considered significant at *P< 0.05.*

Data visualisation was done in the R statistical package (version 4.3). For easier visualisation of metabolite and gene expression data, the control measurements at the beginning of the experiment (**CB**) and control at the end of the experiment (**CE**) were pooled as **C**, as there were no statistically significant differences between the two groups.

## Results

### Relative gene expression

**Figure 1:**
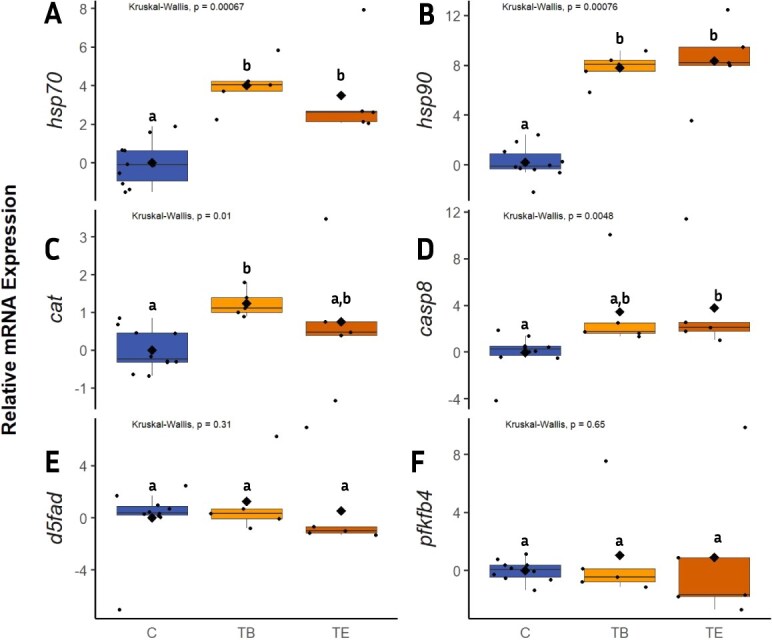
Boxplots showing relative mRNA expression in brown trout liver samples for the control group at 9 °C (**C**) and the 20 °C treatment groups at the beginning (**TB**) and end (**TE**) of the experiment. (A) heat shock protein 70 (*hsp70*), (B) heat shock protein 90 alpha (*hsp90aa1*) (C) catalase (*cat*), (D) caspase 8 *(casp8*), (E) delta-5 fatty acid desaturase (*d5fad*), and (F) 6-phosphofructo-2-kinase/fructose-2,6-biphosphatase (*pfkfb4*). Data are presented as log₂ fold change relative to the control group. Sample number (n) = 5 for TB and TE; n = 10 for C (5 samples from CB and CE were pooled). Letters indicate significant differences between groups (*P < 0.05),* and the black diamond represents the mean.

The *Kruskal Wallis H* test revealed significant differences across groups for the genes *hsp70*, *hsp90aa1*, *cat*, and *casp8*. The genes were upregulated relative to the control (C), especially at the beginning of the treatment (TB). In contrast, the expression of genes *d5fad* and *pfkfb4* showed no significant difference (Fig. 1).

### Energy metabolism

**Figure 2:**
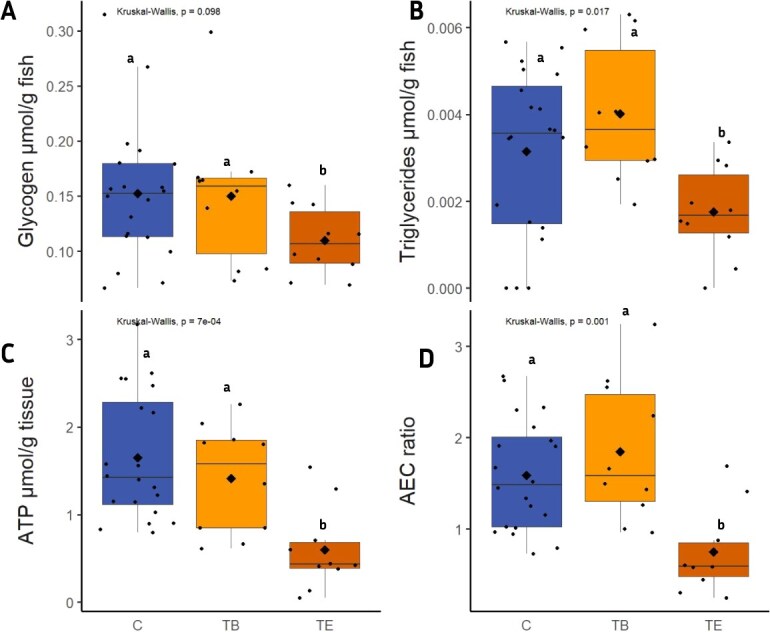
Boxplots showing brown trout’s liver glycogen (A), triglyceride (B), and ATP (C) concentration, and the calculated adenylate energy charge (D), for the 9 °C control group **(C)**, and the 20 °C treatment groups at the beginning **(TB)** and at the end of the experiment **(TE)**. Sample number (n) = 10 for TB and TE; n = 20 for C (10 samples from CB and CE were pooled). Letters indicate significant differences among treatments (*P* < 0.05), and black diamonds represent the mean.

A *Kruskal Wallis H* test showed a significant decrease in liver glycogen, triglyceride, ATP and adenylate energy charge from C and TB to TE (Fig. 2). Data on AMP and ADP, which were used for calculation of adenylate energy charge, are reported in a supplementary file.

### Physiological parameters

The physiological parameters of brown trout are shown in Table 2. An analysis of variance (ANOVA) revealed significant differences in the body mass of fish across the groups, notably showing a 43% suppression of growth in the treatment group compared to the control. There was no significant difference in the hepatosomatic index (HSI) across groups. There was also a significant difference in the fat content of the viscera (FCV) across the groups, with a 70% reduction in the FCV between TB and TE. The survival rate at the end of the experiment for the treatment group was 98%.

**Table 2:** Physiological parameters

Physiological parameter	9 °C, 1d (CB)	9 °C, 21d (CE)	20 °C, 1d (TB)	20 °C, 21d (TE)	Physiological Effect
Body mass(g)	3.5 ± 0.19^a^	5.5 ± 0.16^b^	3.7 ± 0.22^a^	4.3 ± 0.22^c^	43 % Suppressed growth (CB-CE vs TB-TE)
Hepatosomatic index	1.49 ± 0.06^a^	1.54 ± 0.08^a^	1.36 ± 0.08^b^	1.26 ± 0.09^c^	7.4 % Decrease (TB vs TE)
Fat content of viscera (g)	13.0 ± 1.64^a^	8.9 ± 0.67^a^	10.3 ± 0.99^a^	3.0 ± 0.46^b^	70 % Decrease (TB vs TE)
Survival	100%	100%	100%	98%	An average of 99.5% survival across all treatment groups

Table 2 presents data on body mass, hepatosomatic index (HSI), Fat content of the viscera, survival, and physiological effects in brown trout kept at 20 °C for 21 days, compared to a control group at 9 °C. Measurements were taken on day 1 and day 21, with 10 fish per sampling point. Values are presented as means ± SEM, and significant differences (*P < 0.05*) are marked with different superscript letters in the table.

## Discussion

In this study, we demonstrate the effect of elevated temperature on brown trout at the molecular, metabolic, and physiological levels, highlighting the physiological trade-offs of acclimatisation, such as reduced growth, energy storage, and cellular energy status. To our knowledge, no studies have previously investigated the effects of temperature across these multiple levels to understand the physiological and ecological implications on brown trout.

### Cellular stress responses

Heat shock proteins (HSPs) are highly conserved molecular chaperones that are induced to maintain homeostasis and cell function and protect the cells from structural damage caused by different environmental stressors, including elevated temperature (Jeyachandran *et al*., 2023). They play an important role in the folding of synthesised polypeptide chains, as well as in the refolding and degradation of misfolded proteins to prevent them from aggregating and losing their functionality (Hu *et al*., 2022; Kumar *et al*., 2023). As a widely conserved stress response across a wide range of organisms, HSPs are important regulators of thermal stress also in teleosts (Georgopoulos and Welch 1993, Sessions *et al*. 2021).

In the present study, a temperature increase to 20 °C resulted in an upregulation of *hsp70* and *hsp90aa1* relative to the control (Fig. 1A&B). Our results are consistent with those of Beemelmanns *et al*. (2021) in their assessment of temperature stress on Atlantic salmon. Interestingly, comparing the control to TB and TE, our results showed that the highest expression of the HSPs was in the TB group. These results are also consistent with previous studies, which have indicated that a quick production of HSPs is initiated within hours when warm water begins to damage proteins that are required for the normal functioning of cells (Akbarzadeh *et al*., 2018; Von Biela *et al*., 2023). Our results confirmed that exposing brown trout to 20 °C temperature results in thermal stress, which leads to an upregulation of *hsp70* and *hsp90aa1* as a likely cellular defence mechanism to counteract protein misfolding and degradation.

### Oxidative stress response

Increasing temperature also results in a decrease in the solubility of oxygen in water (Levin & Breitburg, 2015). Additionally, increasing temperature leads to an increase in microorganism activities, including respiration and the bacterial degradation rate of organic matter, both processes consuming oxygen. Furthermore, when organisms, especially poikilotherms, are exposed to heat stress, reactive oxygen species (ROS) are produced (Luo *et al*., 2024).

High accumulation of ROS leads to tissue damage, DNA and protein degradation, as well as lipid oxidation (Azad *et al*., 2011; Kubrak *et al*., 2011). To prevent the effects of oxidative stress, an organism will mount an antioxidant primary response system through the expression of genes such as *cat* (Sun *et al*., 2020; Zhang *et al*., 2010). The gene *cat* encodes the enzyme catalase belonging to the enzymatic antioxidant defence systems and catalyses the decomposition of hydrogen peroxide in the reaction 2H_2_O_2_ to 2H_2_O +O_2_ (Anwar *et al*., 2024).

In the present study, there was an upregulation of the gene *cat* relative to the control consistent with previous research on the American shad (*Alosa sapidissima*) (Luo *et al*. 2024). However, contrary to the results of Beemelmanns *et al.* (2021) on Atlantic salmon exposed to incremental temperature from 12 °C to 20 °C, they found no significant differences between the control group and the treatment groups. This could be because Atlantic salmon is a hardier species which can tolerate a wider temperature range compared to brown trout. Interestingly, we also observed a slight decrease in *cat* expression at TE (21 days of exposure to 20 °C). Similar results were found by Sherif *et al.* (2024), who reported changes in *cat* expression in tilapia, *Oreochromis niloticus,* exposed to temperature fluctuations over four weeks following a 14-day acclimation period. Similarly, Olsvik *et al.* (2013) demonstrated that *cat* expression in Atlantic salmon was significantly lower at 19°C compared to 17°C and the control (13°C) over 45 days of study. They suggested that this reduction in *cat* expression resulted from decreased mitochondrial ROS production, likely due to a general metabolic decline at temperatures exceeding the species' optimal range.

In contrast, metabolism generally increases with rising water temperatures, leading to elevated oxygen consumption, ROS production, and oxidative stress (Heise *et al*., 2006). In the present study, we believe that brown trout initially exhibited a peak in *cat* expression upon exposure to 20 °C, due to heightened metabolic activity and increased ROS production. However, by day 21, acclimatisation to the elevated temperature resulted in a reduction in *cat* expression, possibly due to the reregulation of metabolic rate and ROS production to the baseline of the fish. In summary, our findings show that brown trout mounted a strong expression of the gene *cat* as a likely cellular response measure to thermal stress. This demonstrates that increased temperature led to oxidative stress in brown trout, necessitating the upregulation of *cat* gene expression relative to the control.

### Apoptotic processes

Caspase-8 (*casp8*) is a proapoptotic gene enacted during environmental stress, including higher temperatures for programmed cell death. Our results showed an upregulation of the gene *casp8* relative to the control (Fig. 1D). Sun *et al*. (2020) also observed an upregulation of *casp8* at elevated temperatures in largemouth bass (*Micropterus salmoides)* in gill and liver samples. However, our results are contrary to earlier bodies of literature, which suggest that antiapoptotic HSPs can suppress the expression of genes related to promoting apoptosis, such as *casp8* (Garrido *et al*., 1999, 2006; Lanneau *et al*., 2008; Mosser *et al*., 2000). In the present study, both *casp8* and HSP genes simultaneously exhibited increased expression relative to the control. Interestingly, similar results of expression of *casp8* and HSPs genes were observed in Atlantic salmon exposed to water temperatures of 12 °C, 16 °C, 18 °C, and 20 °C by Beemelmanns *et al*. (2021). We propose two hypotheses to explain our results. Firstly, this suggests that an increase in temperature to 20 °C leads to prolonged cellular stress that could result in the accumulation of unfolded or misfolded proteins. This accumulation may exceed the repair mechanism of HSPs in brown trout, thereby necessitating the co-expression of *casp8*. Secondly, *casp8* plays a role in innate cellular immune responses (Orning & Lien, 2021), therefore, its expression may be required beyond its involvement in apoptosis. In general, our findings highlight the need for further investigations on the mechanisms, conditions and extent to which *casp8* expression is inhibited in cells by HSPs. In addition, our findings also suggest that elevated temperature results in the activation of apoptotic functions or immune response through the expression of *casp8* in brown trout.

### Energy metabolism & storage energy metabolism

The gene *d5fad* is involved in fatty acyl desaturation and catalyses the synthesis of monounsaturated fatty acids (MUFAs) from saturated fatty acyl-CoAs. Our results revealed no significant differences in the expression of the lipid metabolism gene *d5fad* (Fig. 1E).

These findings contrast with those of Norambuena *et al.* (2015), who observed significant downregulation of genes associated with fatty acid biosynthesis (*fas*), phospholipid fatty acid release (*cpla2*), fatty acyl desaturation (*d5fad* and *d6fad*), and elongation (*elovl2*) in juvenile Atlantic salmon exposed to elevated temperatures. However, the study investigated the effect of water temperature on the regulation of fatty acid metabolism fed different dietary ARA/EPA ratios (arachidonic acid, 20:4n-6/ eicosapentaenoic acid, 20:5n-3). Therefore, there was also an influence of nutrition in their results. Most of the previous research on *d5fad* in fish have also mainly investigated the gene in response to dietary treatments or supplementation (Hong *et al*., 2024; Torstensen *et al*., 2005; Sanden *et al*., 2016). It has been demonstrated that its expression is generally upregulated in response to diets comprising vegetable oils, such as rapeseed and linseed oils, and downregulated in fish-fed diets rich in highly unsaturated fatty acids (HUFA), including eicosapentaenoic acid and docosahexaenoic acid (Thomassen *et al*., 2012; Tocher, 2003). Therefore, the gene may be more sensitive to nutrition than to temperature, this may explain why we observed no significant differences in the present temperature experiment, which was conducted without diet manipulation, in contrast to the study by Norambuena *et al.* (2015).

Studies on the effects of temperature on fatty acid profiles in ectothermic organisms have reported that there is a general increase in unsaturated fatty acids at lower temperatures and an increase in saturated fatty acids at higher temperatures. This is part of ectothermic organisms’ response to counteract challenges of membrane fluidity caused by fluctuating environmental temperatures (Hazel & Williams, 1990). Therefore, it is intuitive that the expression of *d5fad* either reduces or does not change significantly at elevated temperatures, as observed in the present study.

Furthermore, another study on fatty acid profiles as a predictor of species resilience to temperature by Christen *et al.* (2020) showed that narrow temperature tolerance range salmonids, such as Arctic Charr (*Salvelinus alpinus*, 4°C–16°C), had lower amounts of monounsaturated fatty acids (MUFAs), which are key substrates for carnitine palmitoyltransferase (CPT), an enzyme involved in transporting fatty acids into mitochondria for ATP production. It can be hypothesised that brown trout, also a species with a narrow temperature tolerance, may similarly have lower MUFA levels, potentially reducing its energy supply under thermal stress. Since *d5fad* catalyses the synthesis of MUFAs from saturated fatty acyl-CoAs, its expression plays a critical role in maintaining MUFA levels. Therefore, low MUFA levels may be indicative of low expression of *d5fad*, this feedback loop consequently limits the energy available to meet the increasing metabolic demands at elevated temperatures. In the present study, ATP levels significantly decreased at elevated temperatures, as will be discussed later.

We also investigated the mRNA expression of gene *pfkfb4****,*** which controls the level of fructose-2,6-biphosphate and, therefore, is especially important for glucose metabolism-related processes (Kotowski *et al*., 2021). We found no significant differences in mRNA expression of the gene across groups (Fig. 1F). A study by Long *et al.* (2012) on larval zebrafish showed that the fructose metabolism gene *pfkfb4* amongst other genes was highly upregulated in response to cold stress, while higher temperature did not induce a similar response in the gene, highlighting a specific response to cold. Therefore, this may explain why the present study found no significant differences with elevated temperature exposure.

In addition, *pfkfb4* is also more likely to be regulated by nutrition than temperature, as previous studies that have found significant differences in its expression and modulation have mainly involved diet treatments. (Caballero-Solares *et al*., 2018; Juárez *et al*., 2021).

The findings of our study highlight the gap in knowledge regarding the direct effects of temperature on the metabolism genes *d5fad* and *pfkfb4*, which are crucial for the synthesis of metabolic molecules such as fatty acids and carbohydrates. Further research is needed to understand the precise regulatory mechanisms underlying their expression in response to thermal stress independent of nutrition. Such research will provide a deeper understanding of how ectothermic organisms adapt to temperature fluctuations and the potential ecological consequences for these species, including brown trout, in a warming world.

### Energy storage reservoirs and cellular energy status

To investigate the effect of elevated temperature on brown trout energy metabolism, several metabolites were evaluated, including glycogen, triglycerides, ATP, and the adenylate energy charge (AEC), an indicator of the cellular energy status. Our results showed that the metabolites significantly decreased from C to TB to TE (Fig. 2). Similar results have been found in previous studies on several other species including *Labeo rohita* (Covaci *et al*., 2006; Roychowdhury *et al*., 2020), *Trachinotus blochii* (Das *et al*., 2005) and Atlantic salmon (Kullgren *et al*., 2013). The studies attributed the decrease of glycogen, triglycerides and ATP to higher metabolic rates at higher temperatures, which leads to an increase in the demand for metabolites in the storage reserves. Similarly, the calculated AEC ratio of ATP, ADP and AMP significantly decreased, indicating that the proportion of the nucleotides were off balance because of increased temperature. A higher AEC ratio reflects a higher cellular energy status, while a lower ratio indicates a reduced energy state (Rigoulet *et al*., 2020). According to Ivanovici (1980), the ideal range of AEC for healthy organisms is between 0.8 and 0.9, in the present study, the AEC ratio at TE was less than 0.8. This suggests that elevated temperature significantly affected energy metabolites, leading to their decrease, particularly at TE (after 21 days of the experiment), which indicates a disruption in energy metabolism.

The present study also investigated the effects of elevated temperature on the energy storage of brown trout by examining the metric hepatosomatic index (HSI) and quantifying the fat content of viscera. HSI was investigated because it can reflect metabolic changes and is a storage organ for glycogen and triglycerides, which are the liver's primary energy reserves (Rossi *et al*., 2015). Visceral fat is another lipid depot in teleosts. In times of energy demand under stress, lipids are first mobilised from the viscera for energy production (Navarro & Gutiérrez, 1995). Our results showed no statistically significant changes in HSI across treatments. Similarly, no significant changes in HSI were observed in cherry salmon (*Oncorhynchus masou*) exposed to 10°C, 14°C, 18°C, and 22°C for four or eight weeks (Lee & Balasubramanian, 2023). These results may be because HSI is only a rough estimator of an organism's energy storage capacity (Jacquin *et al*., 2019). Therefore, significant changes in the liver's energy storage capacity may not always be detectable through HSI alone. However, a more sensitive analysis of metabolites in the present study showed a decrease in glycogen and triglyceride concentrations, as previously discussed. On the other hand, a significant decrease (*P ≥ 0.05*) in FCV was observed to a magnitude of 70% (Table 2). This indicates that the stored fats were utilised as an energy source to mitigate temperature stress of 20 °C water temperature for 21 days.

### Physiological and ecological implications

Our results show that exposure to 20 °C for 21 days leads to numerous responses in brown trout at the cellular, metabolic, and physiological levels. The upregulation of genes related to cellular stress response (*hsp70*, *hsp90aa1*, *cat*, *casp8*) observed in the present study comes with energetic costs (decrease in metabolites, energy storage). Physiologically, this culminated in the suppression of growth at elevated temperatures; for instance, the weight increase for the treatment group was only 14% compared to the weight increase of 57% for the control group, a difference of 43%. This is consistent with previous studies on other species, such as the Thai pangas *Pangasianodon hypophthalmus* (Islam *et al*., 2019) and the Atlantic salmon (Pfalzgraff *et al*., 2021) and an earlier study on brown trout juveniles (Ojanguren *et al*., 2001). They attributed suppressed growth at elevated temperatures to stress, reduction of appetite and increased energy demand to maintain homeostasis.

The ecological consequences of our findings are dire. In Austria, water temperatures in some brown trout regions have previously exceeded 20 °C, particularly during hot summers (Melcher *et al*., 2013). As we approach 2100, extreme increases in temperature are expected to become more frequent due to climate change (Planton *et al*., 2008). While our results indicate that brown trout can acclimatise to 20 °C, as demonstrated by a 98% survival rate (Table 2) and the activation of cellular stress defense mechanisms through gene expression, these adaptations come with significant costs. The energy demands associated with stress mitigation can reduce growth and impair reproduction, which is particularly energy-intensive in Teleosts (Wootton, 1990). Over time, such physiological trade-offs may lead to lower overall fitness and survival.

Furthermore, this study focused solely on elevated temperature, whereas brown trout in the wild may experience multiple interacting stressors. These include hypoxia, food scarcity, and disease outbreaks, which can compound the effects of thermal stress (McBryan *et al*., 2013; Todgham & Stillman, 2013; Parisi *et al*., 2017). For instance, rising water temperatures have been linked to an increased prevalence of diseases, further threatening brown trout populations (Harvell *et al*., 2002; Vega-Heredia *et al*., 2024). Given the substantial physiological burden imposed by temperature stress alone, the addition of these stressors could further compromise survival.

In summary, while brown trout may exhibit some resilience to rising temperatures, the cumulative effects of multiple environmental stressors will likely lead to a reduction in overall fitness. To avoid this, brown trout will need to migrate to cooler waters, resulting in changes to their distribution. (Filipe *et al*., 2013; Isaak & Luce, 2023).

## Conclusion

The present study supports the hypothesis that elevated temperatures lead to reduced energy reserves and increased expression of genes related to cellular stress response. Given the imminent threat of global warming and evidence of rising water temperatures, the knowledge gained from this study on the physiological responses of brown trout to elevated temperatures is crucial for implementing conservation practices. This knowledge helps evaluate the ecological consequences of rising water temperatures for the species, thereby informing conservation strategies in a warming world. These strategies may include selective breeding for higher temperature tolerance, as suggested by Jones *et al*. (2004), though this would primarily apply to aquaculture, restoration of river connectivity to facilitate migration to higher elevations with cooler waters and creation of refugia from warm water.

## Supplementary Material

Web_Material_coaf025

## Data Availability

All data used in this study is available upon request from the corresponding author.
