# Supplementary material for: Effects of elevated temperature on gene expression, energy metabolism, and physiology in brown trout, Salmo trutta
Source: Conserv Physiol. 2025 Apr 23;13(1):coaf025. doi: 10.1093/conphys/coaf025 (PMC12015096; doi:10.1093/conphys/coaf025)
Supplement: Web_Material_coaf025 [file web_material_coaf025.zip › Supplemetary File.pdf]

Metabolite concentration in brown trout liver in µmol/g tissue

Treatment	Temperature °C	ATP	ADP	AMP	AEC
C	9	1.028	0.627	0.313	0.681657
C	9	2.22	0.492	0.246	0.833671
C	9	1.222	0.583	0.291	0.72209
C	9	0.898	0.682	0.341	0.644977
C	9	2.55	0.479	0.240	0.853319
C	9	1.144	0.457	0.229	0.75
C	9	1.577	0.490	0.245	0.788062
C	9	2.616	0.478	0.239	0.856586
C	9	1.443	0.379	0.190	0.811382
C	9	0.798	0.531	0.266	0.666771
C	9	2.56	0.488	0.244	0.851762
C	9	2.166	0.515	0.258	0.8246
C	9	3.176	0.571	0.286	0.858294
C	9	2.475	0.514	0.257	0.841651
C	9	1.562	0.500	0.250	0.783737
C	9	1.151	0.485	0.242	0.742013
C	9	0.900	0.449	0.225	0.714422
C	9	1.403	0.497	0.249	0.768497
C	9	1.312	0.491	0.246	0.760127
C	9	0.829	0.411	0.206	0.715422
TB	20	1.822	0.52	0.26	0.800154
TB	20	0.847	0.433	0.216	0.710896
TB	20	0.852	0.421	0.211	0.71597
TB	20	0.61	0.405	0.203	0.667077
TB	20	1.86	0.508	0.254	0.806255
TB	20	0.663	0.441	0.22	0.667296
TB	20	2.26	0.5	0.25	0.833887
TB	20	1.807	0.486	0.243	0.80836
TB	20	1.354	0.503	0.252	0.761261
TE	20	0.051	0.425	0.213	0.382438
TE	20	1.296	0.496	0.248	0.756863
TE	20	0.425	0.378	0.189	0.618952
TE	20	0.443	0.382	0.191	0.624016
TE	20	0.412	0.450	0.225	0.586017
TE	20	0.601	0.301	0.151	0.713675
TE	20	1.541	0.505	0.253	0.780122
TE	20	0.709	0.399	0.200	0.694572
TE	20	0.382	0.305	0.153	0.63631
TE	20	0.132	0.254	0.127	0.504873
TE	20	0.601	0.29	0	0.763738
TE	20	1.541	0.911	0.568	1.691828
TE	20	0.709	0.499	0.25	0.880125
TE	20	0.382	0.087	0.191	0.447909
TE	20	0.132	0.127	0.112	0.303159

Concentration of adenosine triphosphate (ATP), adenosine diphosphate (ADP), adenosine monophosphate (AMP), and the adenylate energy charge (AEC) was calculated using the formula = (ATP + 0.5*ADP) / (ATP + ADP + AMP) for the Control (C), Treatment Beginning (TB), and Treatment End (TE).
